# Identification of genomic regions related to early flowering in *Eucalyptus*

**DOI:** 10.1186/1753-6561-5-S7-P53

**Published:** 2011-09-13

**Authors:** Vanusa Socorro Leite, Julio Cesar Santos Otto, Cíntia Helena Duarte Sagawa, Esteban Roberto Gonzalez, Maria Giziane Fagundes, Shinitiro Oda, Celso Luis Marino

**Affiliations:** 1 Departamento de Genética, UNESP, Botucatu, São Paulo, 18618-970, Brazil; 2 Suzano Papel e Celulose, Suzano, São Paulo, 08613-000, Brazil

## Background

The genus *Eucalyptus* is the most important planted tree to the Brazilian economy. Besides being a source of timber, its wood pulp is mostly used for papermaking. There is a considerable interest in its genetic improvement due to the economic importance of the gender to forestry companies. The time required to obtain ideal populations, after selective breeding, is a limiting factor of classical breeding in these species. However, the breeding program of Suzano Papel & Celulose company obtained a plant of *E. grandis* with the early flowering character, whose first flowering took place between 60 and 90 days. This plant was grown and its seeds were collected and germinated. Progeny segregated for early flowering after approximately 60 to 90 days.

## Materials and methods

AFLP markers [1] combined with bulk segregant analysis method (BSA) [2] were used to search for specific DNA polymorphisms in the genome to produce markers which could identify the character at any stage of development. AFLP markers showed polymorphisms based on the distribution of restriction sites and differential amplification of the fragments. The methodology followed the routine protocols for the AFLP technique and BSA method. Contrasting bulks were prepared, one bulk with ten normal flowering plants and another with ten early flowering plants. DNA was extracted and digested using two restriction enzymes, a frequent cutting (*Mse*I) and a rare cutting (*Eco*RI), followed by connection of the adapters.

## Results and conclusion

The pre-amplification and selective amplification were performed to test 32 primer combinations, which were seen in 6% polyacrylamide gel stained with silver nitrate [3]. As a result, 13 primers amplified and showed polymorphic bands. These amplified fragments are candidates to be specific molecular markers but still need confirmation. Hence, new analyses are being conducted using DNA from each individual separately in order to verify its co-segregation with the early flowering phenotype. Moreover, confirming its effectiveness as a marker in these plants, new strategies will be adopted for validation, such as cloning, sequencing and the development of primers for testing in other populations of *Eucalyptus*. These results indicate the feasibility of using AFLP technique to detect polymorphisms, which might contribute to the improvement of the species.
